# An Interaction Theory Account of (Mediated) Social Touch

**DOI:** 10.3389/fpsyg.2022.830193

**Published:** 2022-05-03

**Authors:** Gijs Huisman

**Affiliations:** Human-Centered Design, Delft University of Technology, Delft, Netherlands

**Keywords:** social touch, mediated social touch, interaction theory of social cognition, haptics, enactivism, phenomenology, participatory sense-making

## Abstract

Research on mediated social touch (MST) has, either implicitly or explicitly, built on theoretical assumptions regarding social interactions that align with “theory theory” or “simulation theory” of social cognition. However, these approaches struggle to explain MST interactions that occur outside of a laboratory setting. I briefly discuss these approaches and will argue in favor of an alternative, “interaction theory” approach to the study of MST. I make three suggestions for future research to focus on.

## 1. Introduction

Social touch is a vital form of intersubjective engagement for human beings and plays an essential role in human development (Fotopoulou and Tsakiris, 2017; Cascio et al., 2019). Later in life, social touch is considered important for the communication of affect (Hertenstein et al., 2006), relationship formation and maintenance (Dunbar, 2010), and stress management (Ditzen et al., 2007). Furthermore, it has been suggested that C-Tactile afferent (CT) affective touch receptors, which selectively respond to slow stroking touches, explain sensory effects of touch (McGlone et al., 2014; Schirmer et al., 2022). However, despite substantial progress in social touch research, there remains considerable debate as to how we should conceive of social touch in actual interpersonal interactions. Social touch interactions are often viewed from a sender-receiver perspective (e.g., Fairhurst et al. 2022; see also Schirmer et al. 2022) where the way humans understand each other in social interactions is considered an individual capacity that involves the exchange of signals (see Schirmer et al., 2022). This perspective is in line with two dominant views in social cognition, namely “theory theory” (TT) and “simulation theory” (ST) which entail theorizing about others' mental states or simulating others' mental states, respectively (see Froese and Gallagher, 2012). Despite the relative dominance of these theoretical frameworks, conceptualisations of social touch as a signal in line with TT and ST frameworks do not translate well to interactions outside of the laboratory (see Schirmer et al., 2022).

Moreover, the debate on how to conceive of social touch extends to research into haptic technology that aims to mediate social touch [i.e., mediated social touch (MST); Haans and IJsselsteijn, 2006; Huisman, 2017]. MST research has resulted in many interesting prototypes (for an overview see Huisman, 2017) and there has been a steady increase in empirical studies into the effects of MST (for overviews see Haans and IJsselsteijn, 2006; Van Erp and Toet, 2015; Huisman, 2017). For example, research has investigated the reproduction of CT-like touch through vibrotactile arrays (Huisman et al., 2016), effects of MST on helping behavior (Haans et al., 2014), and on stress reduction (Sumioka et al., 2013). Nevertheless, null findings are also found in the literature (Erk et al., 2015; Willemse et al., 2018), and there have been significant challenges in replicating social touch through technology (Haans et al., 2014; Ipakchian Askari et al., 2020b). Moreover, effects of MST are strongly dependent on contextual factors (Ipakchian Askari et al., 2020a) that make generalisation difficult. Finally, there are arguments to be made for the field of MST to be experiencing a moment of crisis, which calls for rethinking MST in terms of its social and sensory aspects (Jewitt et al., 2021).

To address the issues outlined above we need new ways to conceive of social touch (e.g., Schirmer et al., 2022), which, I argue, requires us to re-examine the theoretical assumptions underlying our understanding of social cognition with respect to social touch. Here, I argue that we should move away from individualistic theories of social cognition that, implicitly or explicitly, underlie thinking about social touch and MST in many cases, and move toward embodied theories that put a strong emphasis on interaction as playing a central and sometimes constitutive role in how humans understand each other (De Jaegher et al., 2010). This theoretical shift in the way we conceive of social touch can help drive forward a more fruitful research agenda for (mediated) social touch. In the conclusions to this article I provide some initial suggestions for how to furnish such an agenda with the aim of opening up new vistas for research to explore.

## 2. Social Cognition as Interaction

Research on social cognition is concerned with the question of how, on a daily basis, humans are able to understand each other, and this question has been mainly approached from TT and ST perspectives (Froese and Gallagher, 2012; Gallagher, 2020). In TT, social cognition is conceived of as an inferential process based on common-sense, “folk” psychology where the outcomes of theorizing about others' hidden mental states are attributed to the other person (Malle, 2005). In ST, it is supposed that we use our own neural circuitry and mental capacities, including a mirror neuron system (Gallese et al., 2004), as an internal model to simulate the mental states of others. The outcome of this simulation process is then attributed to the other person. Note, that there are different versions of both theories and that hybrid approaches, combining aspects of both TT and ST, also exist (Frith and Frith, 2010).

Taken together, both TT and ST approaches consider social cognition as a reflective, third-person, observation-based process, which is about two (or more) minds, inferring, simulating, or doing a combination of both, to hypothesize about each other's hidden, internal mental states (Gallagher, 2020, p.72). It is an individual process that is driven by sub-personal mechanisms and is considered to apply universally to how people understand each other in social situations (see Froese and Gallagher, 2012; Gallagher, 2020). Both TT and ST approaches can be characterized by methodological individualism and neuro-reductionism (Froese and Gallagher, 2012). The former refers to the way social cognition is studied mainly from the perspective of individuals and their capacities outside of actual social interactions. The latter indicates that the explanation for social cognition needs to be sought in either neural mechanisms or modules (Leslie et al., 2004), or mirror neurons (Gallese et al., 2004), rather than in first-person experience (Froese and Gallagher, 2012).

Despite the wide-spread application of TT and ST in cognitive science, both theories have been criticized for not offering proper explanations for how we engage with each other socially on a day-to-day basis (De Jaegher et al., 2010; Froese and Gallagher, 2012; Gallagher, 2020, Chapter 4). Discussing these criticisms in-depth is beyond the scope of this article, but the most important aspects of the critique for the current argument relate to a focus on the individual that does not explain social interactive processes well, and a dismissal of first-person phenomenological experiences as important, in favor of a focus on neural mechanisms (De Jaegher et al., 2010; Froese and Gallagher, 2012; Gallagher, 2020, Chapter 4).

An alternative to TT and ST is “interaction theory” (IT; Froese and Gallagher, 2012; Gallagher, 2020). IT posits that social cognition cannot be explained fully by only considering individual participants, but that the dynamical, embodied process of interaction is central (De Jaegher and Di Paolo, 2007; De Jaegher et al., 2010; Froese and Gallagher, 2012; Chemero, 2016; Gallagher, 2020). In IT, minds are conceived of in enactive terms (Varela et al., 2016), and IT incorporates ideas from Gibsonian ecological psychology^1^, such as social affordances (Heft, 2020). Minds are not localized in the brain but span brain, body, and environment; they are physically embodied, enacted in interaction, environmentally embedded, and extended (see Newen et al., 2018), and, thus, are not something that is inaccessible and hidden away in the brain or exclusively generated by brain states. IT builds on phenomenology [a detailed overview of the history of phenomenology and its influences on (cognitive) science is provided by Käufer and Chemero (2021)] in arguing that social cognition is dependent on direct perception without mediation by theory or simulation (De Jaegher, 2009; Krueger, 2018). Social understanding depends on, and is sometimes constituted by De Jaegher et al. (2010), immediate real-time interactions with others (Froese and Gallagher, 2012, p.441). In IT, following the definition of De Jaegher and Di Paolo (2007), social interaction is defined as:

a mutually engaged and co-regulated interaction between at least two autonomous and cognitive agents where the co-regulation and the interactive behaviors mutually affect each other, such that the interaction process constitutes a self-sustaining organization in the domain of relational dynamics (Froese and Gallagher, 2012, p.441).

Our embodied, interactive behaviors (which include movements, facial expressions, vocalisations, as well as touch) are always already situated in a social setting that involves cultural practices, social norms, and social roles (Gallagher, 2020). We do not need to theorize or simulate others' mental states because we can understand others through their embodied, interactive behaviors in context, and we respond with our own behaviors, to which they then respond with their own behaviours, and so forth. This co-regulated process is not reducible to mechanisms within each individual but can only be understood by considering the two (or more) dynamically coupled autonomous agents (De Jaegher and Di Paolo, 2007). Thus, we actively participate in generating shared meaning through embodied interactive behaviors (Froese and Gallagher, 2012; Gallagher, 2020, p.104). Here, interaction is the solution to social understanding, not a problem to be solved through theorizing or simulating (De Jaegher, 2009).

A conception of social cognition in IT terms is better able to explain how we understand each other in day-to-day interactions, aligns better with developmental evidence (e.g., Muir, 2002; Buttelmann et al., 2009), takes phenomenological and enactive research into account (e.g., Varela, 1996; Froese and Fuchs, 2012), and considers the holistic nature of brain-body-environment systems (for a detailed discussion of these points see Froese and Gallagher, 2012). Importantly, IT does not claim universality; in some cases, third-person deliberation about an other's mental state my indeed be how one understands another. However, these are the rare exceptions (De Jaegher and Di Paolo, 2013; Gallagher, 2020).

## 3. An Interaction Theory Account of (Mediated) Social Touch

Social touch is most often considered from a “sender and receiver” perspective (see Héron et al., 2021), that involves specific roles such as “communicator” and “recipient” (Jones and Yarbrough, 1985, p.20), or indeed “sender” and “receiver” (Fairhurst et al., 2022, p.57). In other words, social touch involves one person encoding a message that is then to be decoded by another person. For example, Hertenstein et al. (2006)'s definition of tactile communication involves transmission of “one's perceptions, thoughts, and feelings to another” (Hertenstein et al., 2006, p.8). On a more recent account, Fairhurst et al. (2022) discuss a signal-based sender and receiver communication model of affective touch, which they argue allows for the separate investigation of factors that impact the “perceived/decoded experience at the level of the receiver” (Fairhurst et al., 2022, p.55). These quotations suggest an observational, third-person stance toward social interaction that aligns with a TT or ST perspective on social cognition. In addition, the central role of the CT system in many conceptualizations of social touch aligns with a more neuro-reductionist perspective where sub-personal processes (i.e., CT afferents “coding for” social touch) are deemed important in explaining social understanding through touch (McGlone et al., 2014; Fairhurst et al., 2022).

Similar observations can be made with regards to MST. In their review paper of MST research, Haans and IJsselsteijn (2006) argue that social touch is symbolic and should be thought of in terms of sender and receiver. Van Erp and Toet (2015) provide a more sub-personal, brain-focused explanation of social touch in saying that “‘Social touch' is what the brain makes of these [pressure, vibration, stretch, and temperature] stimulus characteristics (sensations)” (Van Erp and Toet, 2015, p.7). Elsewhere, I have provided a similar explanation of social touch in arguing for “a more cognitively involved process” to derive meaning from social touch (Huisman, 2017, p.393).

At the same time, these works do consider social touch to be bidirectional and reciprocal in nature (Muir, 2002; Hertenstein et al., 2006; Fairhurst et al., 2022), and most researchers agree that the context in which social touch occurs is important (Jones and Yarbrough, 1985; Hertenstein, 2002; Saarinen et al., 2021). In their definition of tactile communication, Hertenstein et al. (2006) remark that social touch is “almost always bidirectional and contingent” (Hertenstein et al. 2006, p.8; see also Muir 2002).

Similarly, Fairhurst et al. (2022) reserve a central role for bidirectionality, reciprocity, and the dynamic nature of touch in their communication model. Note, that these aspects align closely with an IT framework focusing on interaction, and less so with frameworks that focus on individual capacities. Thus, if we consider aspects such as bidirectionality and reciprocity to be important, and I argue we should, an IT framework may be better suited to explain social touch interactions.

On an IT account of (mediated) social touch, social touch is not about a sender “composing” (through a deliberate process of either inference or simulation) and sending a message to a receiver, who then engages in an inferential or simulation process to decode the meaning of a touch. Rather, social touch is a co-regulated process between two (or more) autonomous agents who actively participate in the generation of the meaning of the touch in interaction. This interaction is always already part of a context; even touches in lab studies are situated in a “lab setting” with specific social roles and norms. This context operates as a scaffold for the meaning and significance of actions and their expressive movements (Gallagher, 2020, p.165). We do not need to infer or simulate someone's mental states when hugging them at a funeral or wedding, for example; the meaning of the hug is scaffolded by the respective contexts, and is enacted through co-regulated bodily actions (including verbal and linguistic actions Di Paolo et al., 2018). Importantly, the autonomy of the agents in the interaction needs to be maintained (De Jaegher and Di Paolo, 2007; Froese and Gallagher, 2012), and the interaction process itself can take on an autonomy of its own, such as in the case of a handshake where both “shakers” do not let go of each others' hands, maintaining the interaction perhaps for longer than both interactants would like (see De Jaegher and Di Paolo, 2007, p.496 for an example involving kisses). If the autonomy of one agent is somehow reduced, or removed completely, such as in cases of coercion, we would no longer be speaking of a social interaction (De Jaegher and Di Paolo, 2007; De Jaegher et al., 2010). This would also be the case for, for example, transgressive touches, including physical harassment or in extreme cases assault, where an agent does not have (full) autonomy within the interaction. Such transgressive touches would on most conventional accounts be considered social touch along the same lines as a hug (e.g., “systematic changes in another's perceptions, thoughts, feelings, or behavior as a function of another's touch” Hertenstein et al., 2006, p.8). On an IT account where the autonomy of agents matters, this is not the case. Instead, a co-regulated, dynamical process of enacting meaning by autonomous agents, which inherently involves bidirectionality and reciprocity, is what defines social touch on an IT account.

## 4. Discussion and conclusions

In this article I have provided an IT alternative to the dominant TT and ST views on social touch and MST. Here, I make three suggestions that could help shape an IT research agenda for (mediated) social touch.

### 4.1. Active Touch Exploration Rather Than Passive Touch Reception

From an IT perspective (social) touch, is considered as an active, dynamic sense (Carello and Turvey, 2017; Ratcliffe, 2018; Travieso et al., 2020). Suggestions for a stronger focus on the dynamic nature of touch have also been made for haptic technology research, in line with embodied and enactive approaches (Gillespie and O'Modhrain, 2011) and an interactive approach to MST including the use of dynamic haptic feedback (see also Huisman et al., 2021), has been put forth by Héron et al. (2021).

In line with these works, an IT approach to MST should consider the design of tools that enable active exploration through touch, rather than focus on passive touch reception where the recipient of a touch has strongly reduced agency and the focus is mainly on touch sensations (e.g., Huisman et al., 2016). The concept of “augmented sense-making” (Froese et al., 2011b) can be helpful in this regard. Augmented sense-making draws on the enactive approach and refers to devices that are designed to not be the focus of an experience themselves, but that do enable new ways to interactively explore the world. The enactive torch, a haptic navigation device, is an example of an ‘enactive interface' that enables augmented sense-making (Froese et al., 2011b). With these types of ‘active touch devices' the focus is less on the sensation of touch, but more on the use of touch to actively explore, where a user's actions and perceptions mediated through the device are tightly coupled (see Froese et al., 2011b; Froese and Ortiz-Garin, 2020). MST research and the design of MST devices should focus on this active touch component through approaches such as augmented sense-making, because it aligns with the interactive dynamic nature of (social) touch.

### 4.2. Social Interactions Rather Than Individual Responses

Social touch takes place, by definition, during social interactions. However, much work on MST not only puts the focus on touch reception, but conceives of interactions in terms of sender-and-receiver (see Héron et al., 2021), where opportunities for real-time co-regulation are diminished. In some cases, MST is studied in settings where the participant only receives touch and thus no opportunity for actual social touch interaction is present at all (e.g., Jung et al., 2013; Haans et al., 2014).

From an IT perspective, approaches where MST interactions are build around direct interaction where there is no clear distinction between sender and receiver are more fruitful as they more closely resemble naturalistic social touch interactions that revolve around co-regulation processes in which bidirectionality and reciprocity are central. Some devices for MST, such as InTouch (Brave and Dahley, 1997), distributed rope-pulling (Beelen et al., 2013), and coupled haptic knobs (Smith and MacLean, 2007), while not designed from an IT perspective, underscore the dynamical, bidirectional, and reciprocal nature of social touch in a technology-mediated setting that allow for co-regulation to take place.

A paradigm that enables the study of co-regulation in touch interactions is found in a study into haptic perceptual crossing by Auvray et al. (2009). In this paradigm, participants are both present in a minimalist 1-dimensional virtual environment. They both are represented by an avatar controlled with a mouse and they receive haptic feedback when their avatars cross each other in the virtual environment. With several distractors in place, only in situations where there is mutual recognition of each other does the interaction result in a stable state of recognizing the presence of the other (Froese et al., 2020). This perceptual crossing paradigm has been used in a number of studies into technology-mediated social interactions (e.g., Froese et al., 2014; Deschamps et al., 2016; Barone et al., 2020; Hermans et al., 2021) and has potential for the study of MST.

### 4.3. Phenomenological Experience Rather Than Only Outcome Measures

Research on MST has traditionally focused on the effects of MST, and comparatively little attention has been paid to first-person, lived experience in line with the phenomenological foundation of IT (see Froese et al., 2011a). Approaches for studying such lived experience have been developed using haptic interfaces (Froese et al., 2012) and phenomenological interview techniques (Høffding and Martiny, 2016) have already been applied to the study of tactile experiences (Obrist et al., 2013).

With a stronger focus on first-person experiences we also need to recognize that social (touch) interactions take place in context. This necessitates supplementing lab studies with studies taking place in different contexts. Tightly controlled experimental setups might only represent MST as it occurs in the particular situation of a scientific study. Some experimental control may have to be sacrificed in order to provide insights into the lived experience of people using MST devices in diverse contexts (see Saarinen et al., 2021). For example, in a field study by van Hattum et al. (2022), qualitative responses helped shed light on the way MST devices were actually used and experienced by participants over a two-week period of real-world use.

Besides helping understanding of lived experience of MST interactions, a focus on first-person experiences also forces us to consider the fact that lived experiences differ between different people. Rather than focus on sub-personal mechanisms, such as the CT-system (McGlone et al., 2014), a focus on phenomenological experiences would recognize diversity and has the potential to make MST research more sensitive to such diversity in the use of haptic devices for social touch (e.g., see Toro et al., 2020). Different people may enact different meanings through MST; an IT approach to MST would embrace these differences as part of the richness of social interactions.

### 4.4. Conclusions

In this article, I have argued for an IT perspective on MST that conceptualizes social touch as a co-regulated process between two (or more) autonomous agents who actively participate in the generation of meaning of a touch in interaction. The three suggestions for future MST research I provide build on research in ecological psychology, the enactive approach, and phenomenology (see Froese and Gallagher, 2012; Gallagher, 2020), and represent theories and methods that can aid the further development of a more fleshed-out IT perspective on mediated (social) touch. Such a perspective should help shape the design of and research into MST in a way that does justice to the interactive nature of social touch.

## Author Contributions

The author confirms being the sole contributor of this work and has approved it for publication.

## Conflict of Interest

The author declares that the research was conducted in the absence of any commercial or financial relationships that could be construed as a potential conflict of interest.

## Publisher's Note

All claims expressed in this article are solely those of the authors and do not necessarily represent those of their affiliated organizations, or those of the publisher, the editors and the reviewers. Any product that may be evaluated in this article, or claim that may be made by its manufacturer, is not guaranteed or endorsed by the publisher.

## Footnotes

^1^Despite the fact that both ecological psychology and the enactive approach build on phenomenological philosophy there are differences between both these theoretical frameworks. One such difference is in the type of explanation offered. Ecological psychology takes an ontological approach by describing the habitat of a species in terms of the opportunities for action for that species (i.e., affordances), whereas the enactive approach employs an epistemic strategy by starting from self-regulating processes of individual organisms (Baggs and Chemero, 2021). Note, that there is an ongoing discussion on how ecological psychology and the enactive approach could complement each other (e.g., McGann, 2016). Travieso et al. (2020) actually argue that the study of dynamic touch could bridge both frameworks, and it is interesting to consider whether dynamic approaches to *social* touch could do the same for enactive and ecological conceptions of social interactions. Importantly, social interactions in both ecological (Chemero, 2016) and enactive (De Jaegher and Di Paolo, 2007) terms are seen as dynamical processes with social understanding resulting from direct interactions, so there is indeed common ground to build on.
